# Real-Time Aspiration-Enabled Dual Over-the-Wire Microcatheter Enhances Parallel Wire Technique in Chronic Total Occlusion Recanalization

**DOI:** 10.1016/j.jacasi.2026.06.026

**Published:** 2026-09-01

**Authors:** Xin Zhong, Yong Dong, Chenhai Xia, Xiwen Zhang, Changlin Zhai, Bin Nie, Liang Xu, Yujie Zhao, Junbo Ge, Lei Yin, Tao Hu

**Affiliations:** a Shanghai Institute of Cardiovascular Diseases, Zhongshan Hospital, Fudan University, Shanghai, China; b The 7th People’s Hospital of Zhengzhou, Zhengzhou, China; c Xi’an No. 1 Hospital, Xi’an, China; d The Affiliated Huai’an No. 1 People’s Hospital of Nanjing Medical University, Nanjing, China; e The First Hospital of Jiaxing, Affiliated Hospital of Jiaxing University, Jiaxing, China; f The Second Affiliated Hospital, School of Medicine, Zhejiang University, Hangzhou, China; g Xijing Hospital, The Fourth Military Medical University, Xi’an, China

**Keywords:** chronic total coronary occlusion, dual-lumen microcatheter, parallel wire technique

## Abstract

**Background:**

Suboptimal parallel wire technique (PWT) success rates result primarily from progressive subintimal hematoma expansion during procedural maneuvers in chronic total occlusion (CTO) percutaneous coronary intervention (PCI).

**Objectives:**

The aim of this study was to evaluate the procedural outcomes of a novel dual over-the-wire engineered, subintimal hematoma aspiration–enabled dual-lumen microcatheter (DLMC) assisted PWT in CTO PCI.

**Methods:**

One hundred consecutive CTO PCI cases using the PWT were retrospectively analyzed, comparing outcomes between a conventional DLMC (Conv-DLMC) and a H.STARFISH (real-time [RT] aspiration DLMC) assisted PWT.

**Results:**

Reattempt cases constituted 31.0% of all procedures (31 of 100). In the RT aspiration DLMC group, 13 of 50 cases (26.0%) crossed over after Conv-DLMC failure. Hematoma aspiration was performed in 70.0% (35 of 50) of the RT aspiration DLMC group and in 100% of cases (13 of 13) switched from failed Conv-DLMC attempts. PWT success rates were significantly higher with RT aspiration DLMC (98.0% [49 of 50]; 95% CI: 89.4%-99.9%) vs Conv-DLMC (78.0% [39 of 50]; 95% CI: 64.0%-88.5%) (*P* < 0.001). Although median PWT duration showed no significant difference (RT aspiration DLMC vs Conv-DLMC: 22.0 minutes [Q1-Q3: 10.8-51.0 minutes] vs 23.5 minutes [Q1-Q3: 12.0-36.3 minutes]; *P* = 0.735), prolonged PWT attempts (>45 minutes) occurred more frequently in the RT aspiration DLMC group than the Conv-DLMC group (30.0% [15 of 50] vs 12.0% [6 of 50]; *P* = 0.027), with a maximum duration of 147 minutes recorded in the RT aspiration DLMC group. Meanwhile, the Conv-DLMC group required a higher rate of strategy switching to retrograde approach (4.0% [2 of 50] vs 22.0% [11 of 50]; *P* = 0.007). No major complications occurred in either group.

**Conclusions:**

The RT aspiration DLMC was able to improve PWT success rates in CTO PCI and enable prolonged PWT attempts because of its RT hematoma aspiration capability.

Chronic total occlusions (CTOs) are defined as complete coronary artery obstructions characterized by TIMI (Thrombolysis In Myocardial Infarction) grade 0 flow, with an estimated duration exceeding 3 months.^1^ Epidemiologic studies indicate that approximately 15% to 25% of patients with coronary artery disease undergoing diagnostic coronary angiography exhibit at least 1 CTO lesion.^2^, ^3^, ^4^ Historically, CTO percutaneous coronary intervention (PCI) has demonstrated lower procedural success rates and higher complication risks compared with non-CTO PCI. Consequently, CTO recanalization has been termed the “last frontier” of PCI,^5^ reflecting the persistent challenges inherent in recanalizing these complex lesions.

In the early evolution of CTO PCI, the antegrade approach constituted the primary strategy for CTO PCI. Among antegrade techniques, the parallel wire technique (PWT) emerged as a pivotal technique, particularly following the introduction of the dual-lumen microcatheter (DLMC). However, PWT success rates remained suboptimal, primarily because of progressive subintimal hematoma expansion during wire manipulation. This limitation significantly compromised the technique’s efficacy, particularly during prolonged procedures. Over the past 2 decades, advances in devices and techniques have substantially improved the success rates of contemporary CTO PCI.^6^^,^^7^ The integration of the PWT, the retrograde technique, and the antegrade dissection and re-entry technique has yielded worldwide algorithms,^8^, ^9^, ^10^, ^11^, ^12^ accelerating CTO PCI evolution. Nevertheless, the PWT remains a fundamental technique across all algorithms and proves particularly indispensable when retrograde access is unavailable.

Pathophysiologically, hematoma formation constitutes a key determinant of antegrade procedural success. The difficulty of achieving antegrade recanalization increases substantially with progressive subintimal hematoma expansion. Mechanistically informed by this principle, the Stingray system (Boston Scientific) was designed to facilitate hematoma management and true lumen re-entry.^13^ Clinical validation studies have substantiated the efficacy of this hematoma control concept.^14^ Nevertheless, the system’s requirement for specialized equipment has limited its widespread adoption in resource-constrained settings.

Building upon the foundational principle of antegrade hematoma control, we developed an optimized PWT using a novel dual over-the-wire (OTW) DLMC (H.STARFISH, APT Medical) (real-time [RT] aspiration DLMC). This novel DLMC integrates RT hematoma aspiration capability within its dual OTW design, enabling operators to mitigate subintimal hematoma expansion during prolonged PWT attempts without requiring additional equipment exchanges. The aim of this study was to evaluate the clinical efficacy and procedural outcomes of the RT aspiration DLMC–assisted PWT within contemporary CTO PCI practice.

## Methods

### Study population

Between February 2021 and March 2025, patients undergoing the PWT during CTO PCI at 5 high-volume cardiac centers were retrospectively identified. On the basis of the RT aspiration DLMC’s commercial availability date, we consecutively enrolled patients to conventional DLMC (Conv-DLMC) and RT aspiration DLMC groups in a 1:1 matching ratio. The Conv-DLMC group primarily comprised the SASUKE (Asahi Intecc) and Crusade (Kaneka) microcatheters.

All CTO PCI procedures adhered to contemporary clinical guidelines and algorithms.^12^^,^^15^^,^^16^ Procedures were performed by 4 experienced interventional cardiologists, each performing >100 CTO PCIs annually. The study protocol received approval from the medical ethics committees of the 5 hospitals. Written informed consent was obtained from all patients prior to intervention.

### Catheter design and functional mode of the RT aspiration DLMC

**Figure 1:**
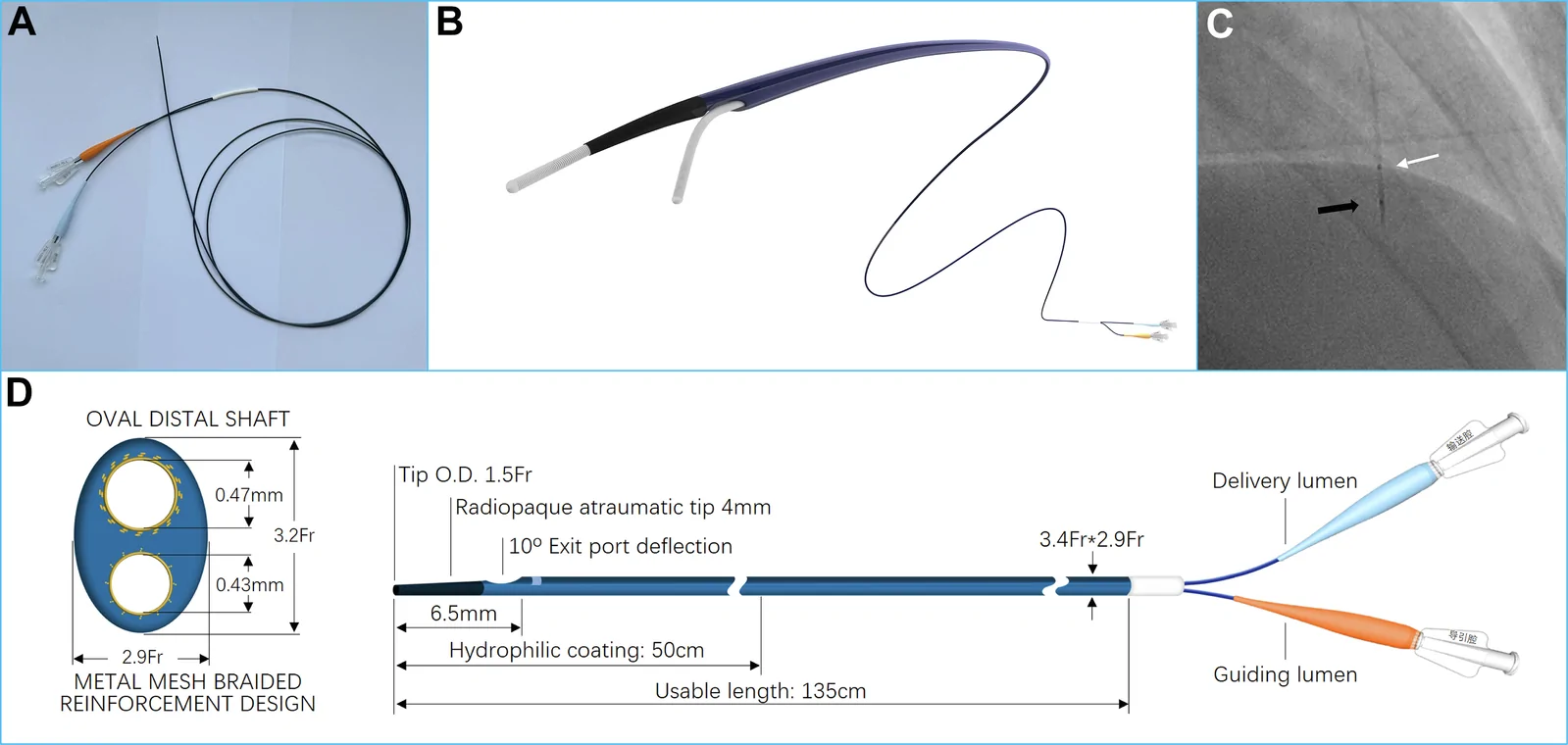
Structural Illustration and Whole Picture of the Real-Time Aspiration Dual-Lumen Microcatheter O.D. = outside diameter.

The RT aspiration DLMC was specifically engineered to optimize the PWT in CTO PCI. This novel microcatheter features a dual OTW lumen design that integrates DLMC functionality with active hematoma aspiration capability. Figure 1 illustrates its physical configuration and schematic design.

The catheter shaft incorporates a composite braid of stainless steel and tungsten wire to enhance trackability through tortuous vasculature. With a 135-cm working length, the distal 50-cm hydrophilic polymer-coated segment improves lubricity, while both lumens feature a polytetrafluoroethylene liner to minimize wire friction. Key dimensions include a maximum outer diameter of 3.4 × 2.9 F, a 1.5-F tip, and inner diameters of 0.43 and 0.47 mm, compatible with 0.014-inch wires. A side port positioned 6.5 mm proximal to the distal tip incorporates a 10° angulation design. The distal architecture features dual radiopaque-marked ports optimized for fluoroscopic visualization during interventions (Figure 1C).

**Figure 2:**
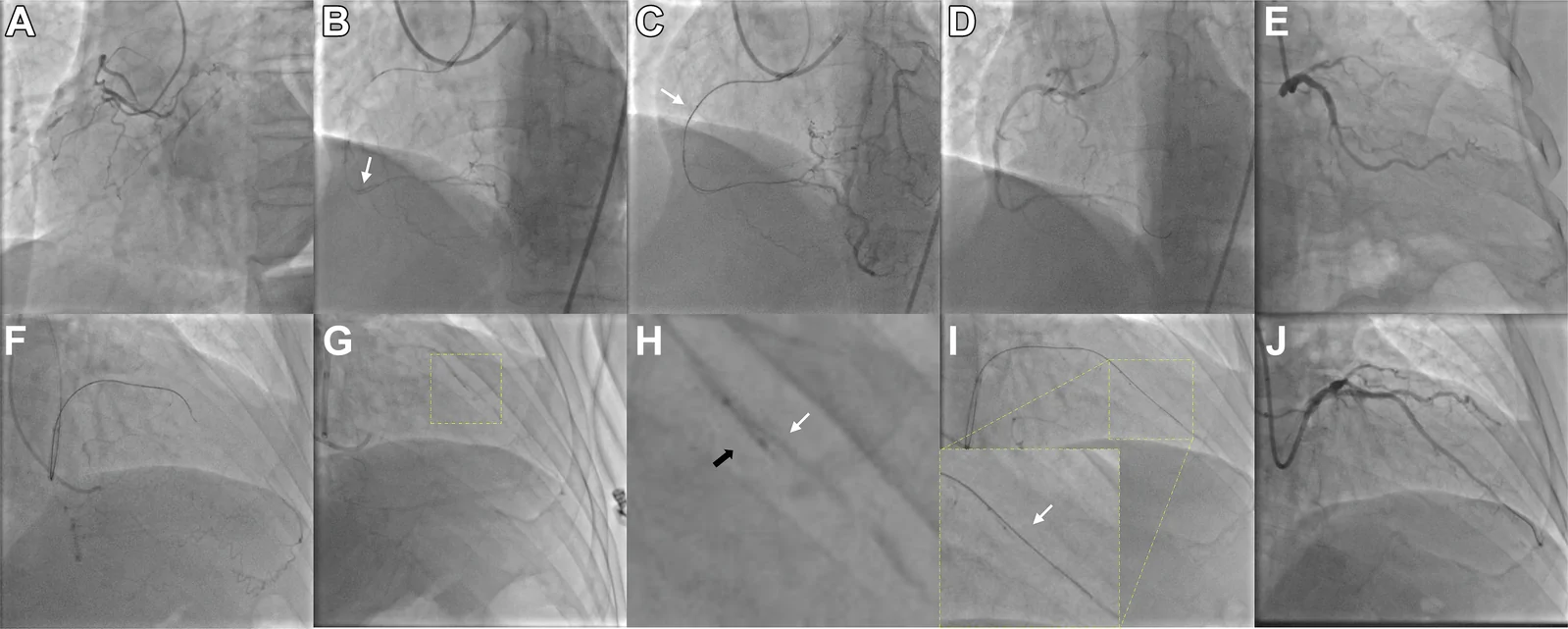
Functional Mode of the RT Aspiration DLMC in Representative Clinical Cases (A) Angiography showed right coronary artery chronic total occlusion (CTO). (B) With real-time (RT) aspiration dual-lumen microcatheter (DLMC) support, contralateral angiography confirmed subintimal wire, distal true lumen above the wire (white arrow). (C) Second wire via RT aspiration DLMC–assisted parallel wire technique (PWT) achieved distal true lumen re-entry. (D) Final angiography after 2 drug-eluting stents. (E) Angiography showed left anterior descending coronary artery CTO. (F) After initial RT aspiration DLMC–assisted PWT, the wire was shown subintimal, and hematoma obscured the distal landing zone. (G) Wire withdrawn, hematoma aspirated via tip port, then contralateral angiography. (H) Magnified view: contralateral angiography showed distal true lumen (white arrow) and RT aspiration DLMC (black arrow) relationship. (I) Under RT tip aspiration, a Gaia Third (white arrow) via the side port entered the distal true lumen. (J) Final angiography after 3 stents.

The RT aspiration DLMC was applied to patients undergoing the PWT during CTO PCI. The standard procedure comprised the following steps (Central Illustration). In step 1, conventional PWT was attempted using both lumens of the RT aspiration DLMC. In step 2, if initial attempts failed, 1 wire (typically from the red lumen) was withdrawn and connected to a vacuum syringe (≥20 mL). Hematoma aspiration was performed to enhance wire-vessel coaptation. In step 3, true lumen re-entry was achieved under angiographic guidance using collateral visualization (ipsilateral or contralateral). When the Conv-DLMC-assisted PWT fails, operators may transition to the RT aspiration DLMC to execute the re-entry sequence (steps 2 and 3). The RT aspiration DLMC features a dual OTW design, offering theoretical flexibility for hematoma aspiration through either lumen. In our practice, aspiration was performed predominantly via the distal tip port, as the specific angulation of the side port provides a more favorable trajectory for the second wire manipulation. In this specific working mode, in which negative pressure is applied through the distal tip port for active aspiration, the RT aspiration DLMC enables a maneuver not achievable with other DLMCs: simultaneous true lumen re-entry with a second wire advanced through the side port, while continuous hematoma aspiration is maintained via the distal tip port. Alternatively, following effective aspiration, both lumens could also be used to accommodate wires for a standard PWT approach. The choice of working mode usually depended on the extent of the subintimal hematoma and the perceived difficulty of achieving control. Figure 2 illustrates the procedural mode of the RT aspiration DLMC in 2 representative cases.

**Central Illustration:**
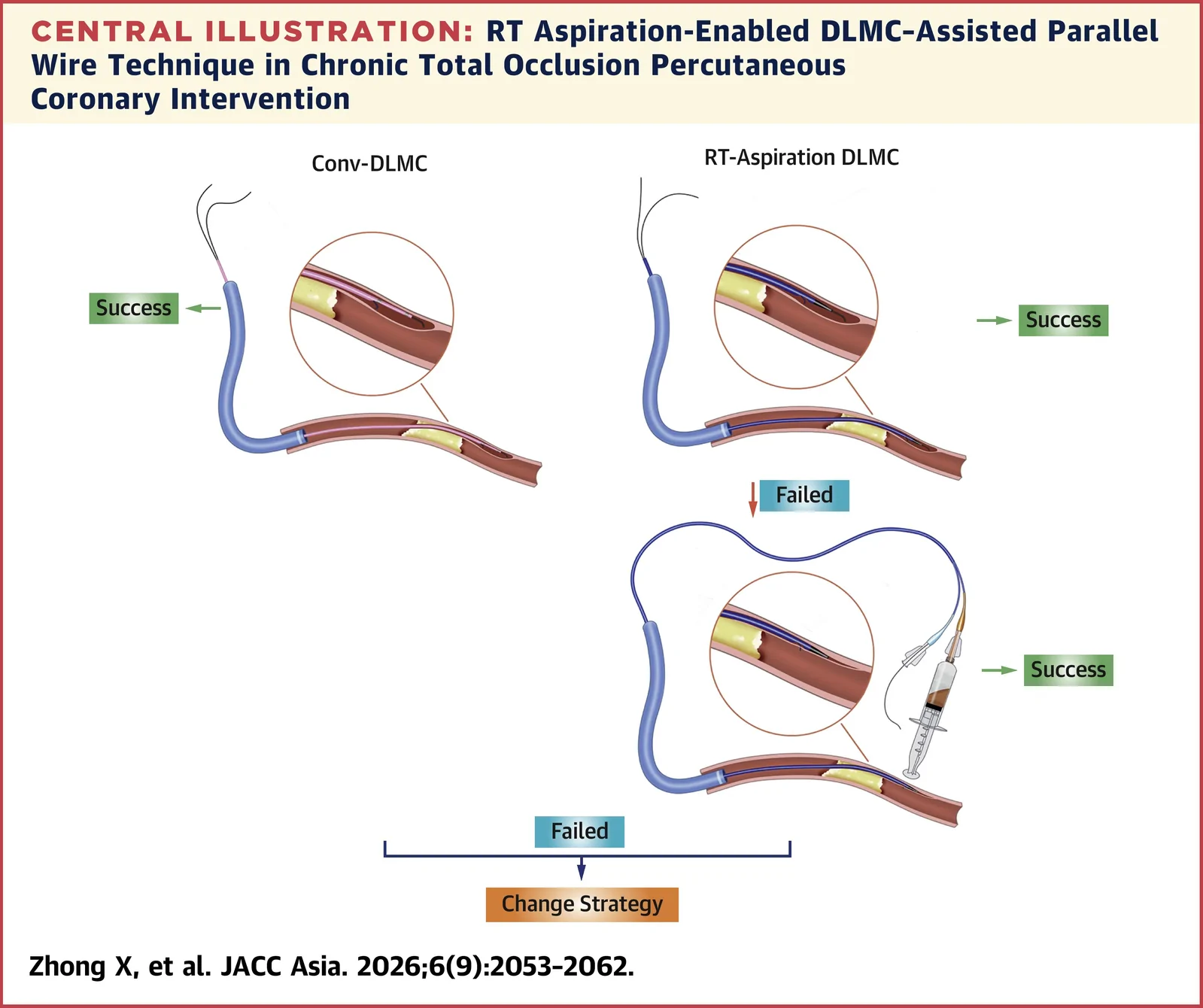
RT Aspiration-Enabled DLMC–Assisted Parallel Wire Technique in Chronic Total Occlusion Percutaneous Coronary Intervention Conv-DLMC = conventional dual-lumen microcatheter; DLMC = dual-lumen microcatheter; RT = real-time.

### Definitions and study endpoints

CTO was defined as TIMI flow grade 0 and a duration of coronary occlusion ≥3 months.^1^ The angiographic indexes examined were the locations of the CTO, stump morphology, calcification at the site of the occlusion (radiopacity present before contrast injection), vessel tortuosity (the presence of at least 1 bend of >45° proximal to the occlusion),^17^ and occlusion length. The J-CTO (Multicenter CTO Registry in Japan) score was calculated according to the previous study reported by Morino et al.^17^ The PWT is defined as a CTO PCI technique in which the initial antegrade wire, having entered the subintimal space, is retained as a positional marker while a second wire is navigated to achieve distal true lumen re-entry. The trajectory of the second wire to the true lumen may traverse either the intraplaque or the subintimal space.

The primary endpoint of this study was the technical success rate of the PWT, defined as successful wire re-entry into the distal true lumen using this specific technique. Secondary endpoints were: 1) PWT attempt duration, measured from DLMC positioning at the target site until successful re-entry, switching to an alternative revascularization strategy, or procedural termination; 2) requirement for rescue retrograde approach; 3) overall procedural and clinical success; 4) major procedural complications; and 5) the incidence of in-hospital major adverse cardiovascular events (MACE).

Procedural success was defined as a restoration of TIMI flow grade 3 in the target vessel and a residual stenosis <30% by visual estimation. Clinical success was defined as achievement of procedural success without in-hospital MACE, which included death of all causes, Q-wave myocardial infarction (MI), and ischemia-driven revascularization. Major procedural complications were defined as donor vessel dissection, vessel perforation,^18^ cardiac tamponade, emergent PCI, and emergent coronary artery bypass grafting. All angiograms and procedural records were reviewed by at least 2 qualified interventional doctors. If there was any ambiguity in the reports or films, the films were reviewed independently by third well-qualified interventional physician.

### Statistical analysis

The normality of continuous data was assessed as needed both visually and using the Shapiro-Wilk test. Normally distributed continuous data are expressed as mean ± SD and were compared between groups using the independent Student’s *t*-test. Non-normally distributed continuous data are reported as median (Q1-Q3) and were compared between groups using the Mann-Whitney *U* test. Categorical variables are reported as frequencies and were compared between groups using the Pearson chi-square test or Fisher exact test as appropriate. For the categorical variables of the J-CTO score (stump morphology, calcification, tortuosity, and occlusion length >20 mm), Cohen’s kappa (κ) values for interobserver variability were calculated. All *P* values were 2-tailed, with a *P* value <0.05 considered to indicate statistical significance. Statistical analyses were performed using SPSS version 20.0 (SPSS).

## Results

### Baseline characteristics

During the study period, 100 consecutive CTO PCI procedures using the PWT were analyzed. Baseline demographic and clinical characteristics are summarized in Table 1. Patients were categorized into the RT aspiration DLMC group and the Conv-DLMC group on the basis of the DLMC used. The mean age was 59.2 ± 12.3 years, and the sample was predominantly male (83 of 100 [83.0%]), with a high prevalence of hypertension (51 of 100 [51.0%]), diabetes (32 of 100 [32.0%]), and history of smoking (42 of 100 [42.0%]). History of prior MI was comparable between the RT aspiration DLMC and Conv-DLMC groups (26.0% [13 of 50] vs 20.0% [10 of 50]; respectively; *P* = 0.476). Reattempt rates did not differ significantly between groups (RT aspiration DLMC vs Conv-DLMC: 30.0% [15 of 50] vs 32.0% [16 of 50]; *P* = 0.829).

**Table 1:** Baseline Characteristics of the Study Patients

	RT Aspiration DLMC (n = 50)	Conv-DLMC (n = 50)	*P* Value
Age, y	58.7 ± 13.4	59.6 ± 11.2	0.710
Male	43 (86.0)	40 (80.0)	0.424
Clinical presentation			
Stable angina	32 (64.0)	31 (62.0)	0.976
Unstable angina	15 (30.0)	16 (32.0)	
NSTEMI	3 (6.0)	3 (6.0)	
Hypertension	27 (54.0)	24 (48.0)	0.548
Diabetes	18 (36.0)	14 (28.0)	0.391
Hyperlipidemia	30 (60.0)	29 (58.0)	0.839
Smoking	22 (44.0)	20 (40.0)	0.685
Prior MI	13 (26.0)	10 (20.0)	0.476
LVEF^a^	55.5 ± 8.2	53.7 ± 8.7	0.316
Reattempted procedure	15 (30.0)	16 (32.0)	0.829

Values are mean ± SD or n (%).

Conv-DLMC = conventional dual-lumen microcatheter; DLMC = dual-lumen microcatheter; LVEF = left ventricular ejection fraction; MI = myocardial infarction; NSTEMI = non–ST-segment elevation myocardial infarction; RT = real-time.

a Data were available for 82 patients.

### Angiographic and procedural characteristics in CTO PCI

Table 2 summarizes the angiographic and procedural characteristics of the CTO PCI procedures. The distribution of CTO target vessels was as follows: left anterior descending coronary artery, 52 of 100 (52.0%); circumflex coronary artery, 12 of 100 (12.0%); and right coronary artery, 36 of 100 (36.0%). Good concordance of interobserver variability was observed for blunt stump (κ = 0.837), calcification (κ = 0.819), vessel tortuosity (κ = 0.858), and occlusion length >20 mm (κ = 0.835). No significant differences were observed in these angiographic characteristics. Specifically, J-CTO scores were comparable between groups (2.1 ± 0.9 vs 2.2 ± 1.1; *P* = 0.584).

**Table 2:** Angiographic Characteristics, Procedural Details, and In-Hospital Clinical Outcomes

	RT Aspiration DLMC (n = 50)	Conv-DLMC (n = 50)	*P* Value
CTO vessel			
LAD	27 (54.0)	25 (50.0)	0.815
LCx	5 (10.0)	7 (14.0)	
RCA	18 (36.0)	18 (36.0)	
Blunt stump	27 (54.0)	30 (60.0)	0.545
Calcification	32 (64.0)	33 (66.0)	0.834
Vessel tortuosity	8 (16.0)	11 (22.0)	0.444
Occlusion length >20 mm	21 (42.0)	22 (44.0)	0.840
J-CTO score	2.1 ± 0.9	2.2 ± 1.1	0.584
IVUS	19 (38.0)	18 (36.0)	0.836
Success of PWT	49 (98.0)	39 (78.0)	0.002
PWT attempt duration, min	22.0 (10.8-51.0)	23.5 (12.0-36.3)	0.735
Bailout retrograde approach	2 (4.0)	11 (22.0)	0.007
Procedural success	47 (94.0)	46 (92.0)	0.500
Clinical success	47 (94.0)	46 (92.0)	0.500
SPM	3 (6.0)	3 (6.0)	1.000
In-hospital major adverse cardiac events			
All cause death	0 (0)	0 (0)	1.000
Q-wave MI	0 (0)	1 (2.0)	0.500
Ischemia-driven revascularization	0 (0)	0 (0)	1.000
Procedural complications	0 (0)	0 (0)	1.000

Values are n (%), mean ± SD, or median (Q1-Q3).

CTO = chronic total occlusion; DCB = drug-coated balloon; IVUS = intravascular ultrasound; J-CTO = Multicenter CTO Registry in Japan; LAD = left anterior descending coronary artery; LCx = left circumflex coronary artery; PWT = parallel wire technique; RCA = right coronary artery; SPM = subintimal or plaque modification; other abbreviations as in Table 1.

Regarding technical parameters, both groups used the DLMC-assisted PWT in the primary antegrade approach. In the RT aspiration DLMC group, intraprocedural conversion from Conv-DLMC to RT aspiration DLMC occurred in 13 of 50 cases (26.0%). Hematoma aspiration was performed in 70.0% of the overall RT aspiration DLMC group (35 of 50) and in 100% of cases transitioning from failed Conv-DLMC attempts (13 of 13). Intravascular ultrasound use was similar between groups. Among the 37 patients who underwent intravascular ultrasound examination, subintimal wire tracking was observed in 19 cases (51.4%), and this proportion was also comparable between the 2 study groups (*P* = 0.873).

In most cases, high-penetration wires were used for final true lumen re-entry. In the Conv-DLMC group, the wire types used were as follows: Gaia Third (Asahi Intecc; 31 of 50 [62.0%]), PILOT 200 (Abbott Cardiovascular; 9 of 50 [18.0%]), Conquest Pro 12 (Asahi Intecc; 6 of 50 [12.0%]), Conquest Pro 8-20 (Asahi Intecc; 2 of 50 [4.0%]), and ULTIMATEbros 3 (Asahi Intecc; 2 of 50 [4.0%]). In the RT aspiration DLMC group, the distribution was as follows: Gaia Third, 33 of 50 (66.0%); Pilot 200, 9 of 50 (18.0%); Conquest Pro 8-20, 5 of 50 (10.0%); and Conquest Pro 12, 3 of 50 (6.0%). The distribution of penetrating wires was similar between the 2 groups (*P* = 0.361).

### Outcomes of the DLMC-assisted PWT and procedure in CTO PCI

**Figure 3:**
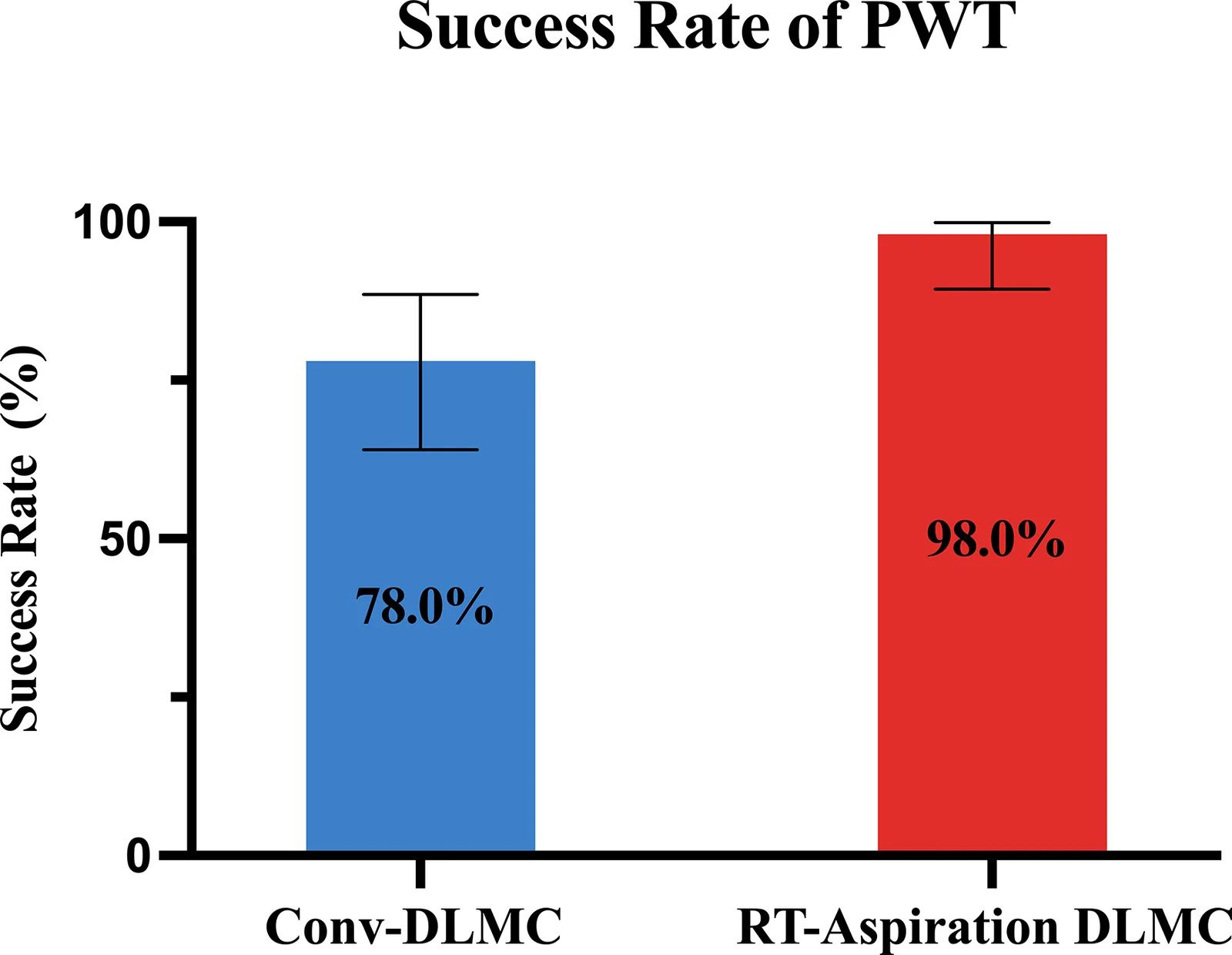
Success Rate of the PWT for the RT Aspiration DLMC and Conv-DLMC Groups The 95% CIs for the success rate of PWT are plotted. Conv-DLMC = conventional dual-lumen microcatheter; other abbreviations as in Figure 2.

Overall, the success rate of the PWT was significantly higher in the RT aspiration DLMC group than in the Conv-DLMC group (98.0% [49 of 50] [95% CI: 89.4%-99.9%] vs 78.0% [39 of 50] [95% CI: 64.0%-88.5%]; *P* < 0.001) (Figure 3). This significant difference was reflected in the strategy switching: a substantially greater proportion of the Conv-DLMC group required a rescued retrograde approach (4.0% [2 of 50] vs 22.0% [11 of 50]; *P* = 0.007). Notably, 3 patients in the RT aspiration DLMC group demonstrated salvage PWT success after unsuccessful retrograde collateral tracking.

**Figure 4:**
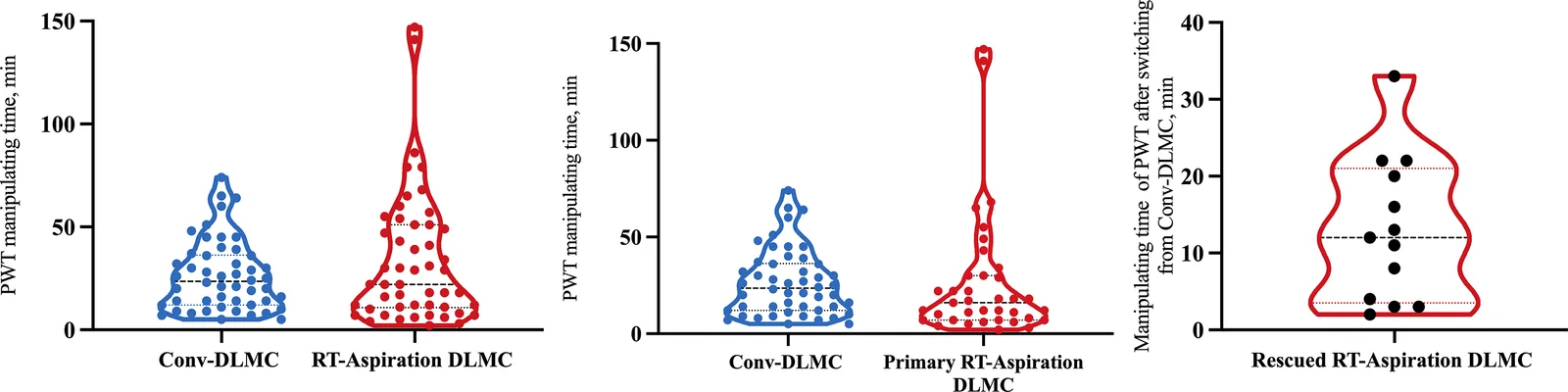
PWT Attempt Duration for the RT Aspiration DLMC and Conv-DLMC Groups Abbreviations as in Figures 2 and 3.

PWT attempt duration is illustrated in Figure 4. Although median PWT duration was comparable between groups (RT aspiration DLMC vs Conv-DLMC: 22.0 minutes [Q1-Q3: 10.8-51.0 minutes] vs 23.5 minutes [Q1-Q3: 12.0-36.3 minutes]; *P* = 0.735) (Figure 4A), prolonged PWT attempts (PWT duration >45 minutes) occurred more frequently in the RT aspiration DLMC group than the Conv-DLMC group (30.0% [15 of 50] vs 12.0% [6 of 50]; *P* = 0.027), highlighting the extended attempt duration within the RT aspiration DLMC group (maximum 147 minutes). This dispersion pattern persisted even after excluding the crossover procedures (conversion to RT aspiration DLMC following Conv-DLMC failure) (Figure 4B). In the crossover subgroup, successful PWT was achieved within a median of 12.0 minutes (Q1-Q3: 3.5-21.0 minutes) postconversion (Figure 4C).

Despite significant differences in PWT success rates, overall procedural success remained comparable between groups (RT aspiration DLMC vs Conv-DLMC: 94.0% [47 of 50] [95% CI: 83.5%-98.7%] vs 92.0% [46 of 50] [95% CI: 80.8%-97.8%]; *P* = 0.617), attributable to effective salvage strategy switching to retrograde approaches. The incidence of investment procedure occurred at identical rates (6.0% [3 of 50]) in 2 groups. In-hospital MACE occurred in 1 patient (1 of 50 [2.0%]) in the Conv-DLMC group following procedural failure, presenting as postprocedural Q-wave MI. No other in-hospital MACE components were observed. Overall complication rates were notably low, with a single minor access-site hematoma (femoral artery) documented in the RT aspiration DLMC group and managed conservatively without intervention.

## Discussion

Focusing on the PWT in CTO- CI, the present study introduced a novel dual OTW designed, subintimal hematoma aspiration–enabled DLMC. Our findings demonstrate the effectiveness and safety of the RT aspiration DLMC–assisted PWT in real-world, multicenter CTO PCI procedures.

### The challenges of the PWT in contemporary CTO PCI

The PWT remains a fundamental antegrade technique in contemporary CTO PCI and is prominently integrated into established procedural algorithms.^9^, ^10^, ^11^, ^12^ Its core principle involves identifying the optimal re-entry point and manipulating a secondary wire to achieve true lumen re-entry from this critical position. However, contemporary parallel wire techniques demonstrate suboptimal success rates.^19^, ^20^, ^21^ The practical implementation of the PWT faces significant constraints, primarily because of progressive subintimal hematoma expansion during prolonged attempts. This pathophysiological process displaces the vessel wall from the wire, rendering targeted puncture mechanically challenging and frequently necessitating PWT abandonment.

Although the Stingray system advanced hematoma management through controlled subintimal access, it lacks RT aspiration capability. Additionally, device-specific requirements and cost barriers have constrained its global accessibility. The RT aspiration DLMC introduces a paradigm-shifting approach by integrating RT active aspiration capability within a Conv-DLMC design. This innovation enables significant hematoma volume reduction during PWT execution, substantially enhancing the feasibility of true lumen re-entry, particularly in cases in which the Conv-DLMC-assisted PWT has failed. Notably, among crossover cases in which Conv-DLMC assisted PWT was unsuccessful (n = 13), rescue PWT was successfully achieved following transition to the RT aspiration DLMC.

### Structure of the RT aspiration DLMC and learning curve

The fundamental innovation of the RT aspiration DLMC resides in its dual OTW design, a structural advancement over Conv-DLMC systems that enables RT hematoma aspiration during wire re-entry attempts. This core mechanism is central to the RT aspiration DLMC’s superior PWT success rates. Operationally, operators usually use the distal (red) port for aspiration and the proximal (blue) lumen for wire manipulation during RT hematoma aspiration. However, the configuration remains adaptable to anatomical demands at the operator’s discretion.

Meanwhile, we identified another dual OTW DLMC, the ReCross (IMDS). The ReCross exhibits notable design divergences, including diametrically opposed side ports with 180° orientation,^22^ which align more closely with the mechanism of the Stingray system. This structural configuration distinguishes it fundamentally from the RT aspiration DLMC, which was specifically engineered to integrate RT aspiration capability while maintaining optimal directional guidance for precise re-entry during the PWT. Furthermore, no studies have been reported on the optimization of PWT using the ReCross DLMC.

Because of the intuitive design, operators reported that the RT aspiration DLMC–assisted PWT exhibited a short learning curve, enabling rapid proficiency acquisition. The procedural steps integrate seamlessly with conventional PWT, while the intuitive aspiration mechanism demands minimal additional training. Despite including cases with prolonged PWT durations (up to 147 minutes), our data set revealed no major procedure-related complications in the RT aspiration DLMC group. These findings support the device’s favorable safety profile, even during extended procedural attempts.

### Advantages of the RT aspiration DLMC: enhanced success rate, optimized workflow, and procedural efficiency

Our data demonstrate a significantly higher PWT success rate with the RT aspiration DLMC vs a Conv-DLMC (98.0% [49 of 50] vs 78.0% [39 of 50]; *P* < 0.001). This superiority is attributable primarily to the RT aspiration DLMC’s capacity for hematoma management. Upon conventional PWT failure, active aspiration through 1 lumen substantially reduces subintimal hematoma volume, facilitating optimal wire–vessel wall coaptation and enhancing true lumen re-entry efficiency. Mechanistically, hematoma expansion during prolonged attempts obscures distal landing zones on angiography. RT aspiration DLMC aspiration restores visualization of: 1) the target landing zone; and 2) the RT aspiration DLMC’s spatial relationship to the distal true lumen, providing critical guidance for precise puncture point identification.

Conventional PWT protocols typically avoid antegrade contrast injection to prevent hematoma exacerbation, especially when retrograde imaging guidance is insufficient. The RT aspiration DLMC overcomes this limitation through RT hematoma control, enabling antegrade contrast administration even in cases with poor retrograde collateral visualization. Device delivery to the target site frequently requires 1.5-mm balloon predilation. Although antegrade balloon dilation carries the risk for hematoma expansion, the RT aspiration DLMC’s robust aspiration capability effectively mitigates this situation, permitting an uninterrupted PWT attempt and antegrade contrast injections if required.

### Procedure time: extended operative window enabled by real-time hematoma management

Analysis of procedure times revealed comparable median PWT durations between the RT aspiration DLMC and Conv-DLMC groups. The mean successful procedure time in the RT aspiration DLMC group was 22.0 minutes. Notably, after excluding cases requiring crossover from Conv-DLMC failure, the mean successful procedure time for the native RT aspiration DLMC group decreased to 16.0 minutes (Q1-Q3: 7.0-30.0 minutes). These findings demonstrate the high efficacy of the RT aspiration DLMC in achieving PWT success. Critically, within the crossover subgroup (initial Conv-DLMC failures), conversion to the RT aspiration DLMC enabled successful PWT completion within finite time frames in which the Conv-DLMC-assisted PWT had proved unsuccessful.

A significantly wider dispersion in procedure times was observed in the RT aspiration DLMC group (Figure 4), including prolonged attempts (maximum 147 minutes). This distribution pattern confirms the device’s unique capacity to maintain procedural feasibility during extended manipulations. Such extended durations are typically unattainable with the Conv-DLMC because of uncontrolled hematoma expansion. Collectively, these temporal profiles highlight the pivotal advantage of the RT aspiration DLMC: maintaining a controlled subintimal environment via active aspiration.

### Potential for extended subintimal tracking

During RT execution of the PWT, the precise course of the wire, whether intraplaque or subintimal, cannot be definitively determined. This distinction can only be determined retrospectively using intravascular imaging modalities. Consequently, some cases involved intraplaque crossing, whereas others followed a subintimal trajectory. Although the RT aspiration DLMC–assisted PWT significantly improves true lumen re-entry success rates, one issue should be noted: successful re-entry occasionally occurred at distal locations, resulting in extensive subintimal tracking in certain cases. The RT aspiration DLMC–assisted PWT facilitates targeted re-entry at radiographically evident points through controlled hematoma aspiration. However, when the re-entry site is excessively distal, operators may ultimately adopt a deferred stenting strategy because of substantial subintimal dissection or big side-branch compromise.^23^^,^^24^ In the present study, the incidence of investment procedure was identical in both the RT aspiration DLMC and Conv-DLMC groups (6.0% [3 of 50]). It is noteworthy that within the scope of contemporary evidence, both intraplaque and subintimal recanalization techniques are associated with comparable mid-term angiographic and clinical outcomes.^25^^,^^26^ Nevertheless, the current sample size precludes definitive conclusions regarding long-term implications, warranting further investigation in larger cohorts.

Consequently, although the RT aspiration DLMC demonstrably enhances PWT success rates, operators should remain cognizant of its potential to produce extended subintimal dissection planes. When long subintimal segments are encountered or anticipated, switching to a retrograde approach should be considered.

### Study limitations

First, this study’s retrospective design and relatively small sample size may constrain the generalizability of our findings. Multivariable adjustment would risk overfitting and unstable estimates; therefore, we opted to present univariate analyses as the primary results. Our findings should be interpreted as exploratory rather than definitive, and the estimated effects may be subject to bias due to imbalances in baseline characteristics between groups. Future larger scale studies are warranted to confirm these findings. Nevertheless, given that contemporary CTO PCI has reached a stage of technical maturation, the aim of the present work was to introduce a novel RT aspiration-enabled dual OTW microcatheter to facilitate PWT during CTO PCI, thereby offering a meaningful enhancement to existing procedural algorithms. Such pioneering investigations remain scarce in the current literature.

Second, all procedures in this study were performed by 4 high-volume CTO operators (each performing >100 CTO PCI procedures annually). Although the RT aspiration DLMC exhibited a relatively short learning curve during its implementation, further validation across a broader range of operators is necessary to confirm its user-friendliness and reproducibility.

## Conclusions

Our results demonstrated that the RT aspiration DLMC significantly improved the success rate of the PWT in CTO PCI. Because of its RT hematoma aspiration capability, extended PWT attempt duration is enabled in antegrade approach.

## Funding Support and Author Disclosures

Dr Hu holds the patents on the H.STARFISH DLMC. All other authors have reported that they have no relationships relevant to the contents of this paper to disclose.
